# Changes to the Employers' Use of Genetic Information and Non-discrimination for Health Insurance in the USA: Implications for Australians

**DOI:** 10.3389/fpubh.2018.00183

**Published:** 2018-06-20

**Authors:** Gemma A. Bilkey, Gareth Baynam, Caron Molster

**Affiliations:** ^1^Office of the Chief Health Officer, Public and Aboriginal Health Division, Department of Health, Government of Western Australia, Perth, WA, Australia; ^2^Genetic Services of Western Australia, Department of Health, Government of Western Australia, Perth, WA, Australia; ^3^Western Australian Register of Developmental Anomalies, Department of Health, Government of Western Australia, Perth, WA, Australia; ^4^Office of Population Health Genomics, Public and Aboriginal Health Division, Department of Health, Government of Western Australia, Perth, WA, Australia; ^5^School of Paediatrics and Child Health, University of Western Australia, Perth, WA, Australia; ^6^Institute for Immunology and Infectious diseases, Murdoch University, Perth, WA, Australia; ^7^Telethon Kids Institute, Perth, WA, Australia; ^8^Spatial Sciences, Department of Science and Engineering, Curtin University, Perth, WA, Australia

**Keywords:** genetic testing, medicolegal, ethics, gene expression, privacy, indigenous health, confidentiality, politics

## Abstract

In the USA, a bill has been introduced to the senate that may jeopardize an individual's rights to privacy and non-discrimination. This piece examines the proposed Preserving Employee Wellness Programs Act (PEWPA), and implications this will have on the use of genetic information. The Act allows for employers to apply financial penalties for health insurance based on genetic information, which raises concerns as the capacity to interpret genetic results is limited by knowledge of the significance of both benign and pathogenic variants. In Australia, genetic information can only be used to determine life insurance, not to stratify health insurance, and any precedent set internationally should raise concerns of the potential for change on the horizon.

The year 2017 saw the introduction of the proposed “Preserving Employee Wellness Programs Act” (PEWPA) to Congress in the United States of America (USA). At its core, PEWPA allows for employers to bypass the employee's rights for privacy of genetic information when requested under a loosely defined guise of a “wellness program.” Such programs are purported to inform and empower employees for health lifestyle choices, and implement targeted health promotion and prevention programs. However, PEWPA enables employers to impose both rewards and penalties for its employees to participate in such wellness programs, such as the ability to increase health premiums as a financial penalty based on a person's genetic data, or more worryingly, a person's non-disclosure of their genetic information. The ability to apply such penalties represents a significant encroachment on an individual's rights under the Genetic Information Non-discrimination Act (GINA), and the Americans with Disabilities Act (ADA). PEWPA provides a loophole to undermine the privacy and nondiscrimination provisions that GINA and the ADA attempt to protect. With access to healthcare tied with employment for most citizens of the USA, the decision for the employee to participate in any employer initiated requests for genetic information and disclosure are complex (1, 2).

Fraught with ramifications for health information privacy and discrimination, the international precedent that may be set in the USA has potentially global implications. At present, genetic testing technology has outrun our ability to interpret the results, culminating in the identification of gene variants of unknown clinical significance. Robust interpretation of genetic information requires extensive knowledge of benign and pathogenic variants. While technology has allowed us to “read” the genome, our genomic literacy is lagging behind, challenging our ability to understand and interpret the many variations of uncertain clinical significance (3). This is particularly, and inequitably, so for Aboriginal and Torres Strait Islander Australians as there is a paucity of reference genomic data for this population (4).

The interplay between genetics and nurture for many of these variants, and how this impacts on penetrance of disease processes is poorly understood. PEWPA seemingly ignores the conundrum that presence of a variant (existence of a genetic mutation) does not necessarily lead to penetrance (realization of a disease). That an employer could apply a financial penalty for simply having the presence of a genetic variant, over realization of the disease is of grave concern. Similarly for an employer to request genetic information, questions both informed consent of genetic testing, and autonomy of health care decision-making for the employee (5).

Furthering understanding of both benign and pathogenic variants relies on the impartation and sharing of genetic data, without fear of discrimination. For potential research participants in the USA, significant consideration would need to be given to the future use of their genetic information, knowing that an employer may request disclosure of such data, and penalties applied for non-compliance. PEWPA therefore significantly risks slowing the rapid progress made toward our understanding of disease and precision medicine, with uncertain global implications for research and innovation in genomics (2).

In Australia, two key pieces of Commonwealth legislation provide protection to the public against discrimination based on genetic information from insurance agencies and employers. These are the *Disability Discrimination Act (DDA) 1992*, and the *Human Rights and Equal Opportunity Commission Act (HREOC) 1984*. More specifically for health insurance in Australia, the *Private Health Insurance Act (PHIA) 2007*, prohibits the stratification of individuals' premiums based on health status (6). Furthermore, the *Workplace Relations Act (WRA)* prohibits discrimination on a range of grounds in terminating employment. These Acts currently prohibit discrimination on the basis of genetic information, however this assumption holds true only as long as these Acts are in force in their present form (7). Currently, in accordance with the *DDA* (s.46), the insurance industry in Australia allows for genetic information to be used for determination of life insurance, but not for health insurance, provided compliance with the terms of the *PHIA* (s.55.5) (6, 8). The ability for life insurance companies to ascertain genetic information in Australia has been documented to deter uptake of genomic services in at-risk individuals, and it is noted that there has been no review of legislation since 2005 (9). In recognition of the complexities in genomic medicine, the Commonwealth Department of Health recently published the National Health Genomics Policy Framework, which prioritizes the responsible collection, storage, use, and management of genomic data, as well as recognizing the need for a coordinated approach to the ethical, legal, and social issues inherent in this space (10).

With the landscape in the USA providing new uncertainty in the protection of genetic information, the future implications for public policy and comparable legislation in Australia, as well as other countries, must be considered. The propensity for global policy transfer has been demonstrated through the adoption of the “work for welfare” programs, which commenced in the United States, subsequently catalyzing uptake in other industrialized countries such as Britain, Australia, and Sweden (11, 12). Similarly, the addition of medicinal cannabis to the political agenda in Australia and around the world can also be aligned with social pressure arising from legalization in other jurisdictions (13). If PEWPA is realized, the overarching implications for research, development, discrimination, and privacy are portentous and are likely to reverberate beyond our northern hemisphere neighbors.

## Author contributions

GAB and GB have made a substantial, direct and intellectual contribution to the work. CM assisted in the concept development and initial stages. All authors have approved it for publication.

## Conflict of interest statement

The authors declare that the research was conducted in the absence of any commercial or financial relationships that could be construed as a potential conflict of interest.
